# Latent Profiles of Maternal Parenting Stress Moderate the Relationship Between Overparenting and Young Children’s Social–Emotional Competence

**DOI:** 10.3390/ejihpe16100165

**Published:** 2026-10-15

**Authors:** Wen Xiao, Suqun Liao

**Affiliations:** School of Educational Science, Shaoguan University, Shaoguan 512005, China; xws@sgu.edu.cn

**Keywords:** parenting stress, psychological well-being, parenting practices, social–emotional competence, latent profile analysis, early childhood

## Abstract

This study examined how maternal parenting stress and overparenting are associated with young children’s social–emotional competence using both variable-centered and person-centered approaches. A total of 445 mothers of young children completed measures of parenting stress, overparenting, and children’s social–emotional competence. Variable-centered analyses showed that positive experience was associated with children’s social–emotional competence through overparenting. Latent profile analysis identified three maternal parenting stress profiles based on different combinations of positive experience and strain, namely disengaged, engaged–high-strain, and engaged–low-strain. Children of mothers in the engaged–low-strain profile showed the highest levels of social–emotional competence, whereas those in the disengaged profile showed the lowest levels. Further analyses showed that the association between overparenting and children’s social–emotional competence differed across maternal stress profiles. This association was significantly stronger among mothers with high positive experience and low strain than among mothers experiencing high strain. These findings suggest that the association between overparenting and children’s development depends on mothers’ broader parenting stress profiles. They also highlight the importance of considering the heterogeneous and two-sided nature of maternal parenting stress when examining its implications for children’s social–emotional competence.

## 1. Introduction

Persistently low fertility has become a defining demographic trend across many societies, especially in East Asia. As family sizes shrink, parents pay closer attention to each child’s development. Parenting stress, particularly among mothers, has become a public health concern. High parenting stress can lower parents’ intentions to have more children (Chen et al., 2024). It is therefore urgent to identify family factors that support children’s healthy development. In China, rapid social change and rising educational expectations push parents to maximize their children’s success (Di et al., 2026). These pressures have led to more intensive parenting and greater parental involvement in children’s daily lives (Novoa et al., 2025). Maternal parenting stress may show up not just as emotional distress but as more controlling or intrusive parenting, such as overparenting practices (Schoppe-Sullivan et al., 2024; Slobodin et al., 2020). However, whether overparenting links maternal parenting stress to young children’s social–emotional competence remains unclear.

Social–emotional competence is the ability to use emotional information to solve problems, establish positive peer relationships, and behave prosocially (Rose-Krasnor & Denham, 2011). Children with higher levels of social–emotional competence make a smoother transition from kindergarten to primary school and tend to have better mental health and social functioning later in life (Domitrovich et al., 2017). Parenting stress has consistently been identified as a key risk factor for young children’s social–emotional competence. Parenting stress arises when the demands of child-rearing exceed the psychological and material resources available to parents (Abidin, 1990). It tends to be particularly pronounced during the preschool years (Anthony et al., 2005). Prolonged parenting stress depletes parents’ psychological resources and reduces their capacity for sensitive, responsive caregiving (Fang et al., 2024). This puts children at greater risk of emotional dysregulation and social withdrawal (Santelices & Cortés, 2022). Parenting stress has also been associated with lower children’s social–emotional competence and greater internalizing and externalizing problems in early childhood (Sourander et al., 2025). Yet the specific parenting behaviors that carry these effects remain understudied (Masarik & Conger, 2017).

Although parenting stress is often conceptualized as a unidimensional construct (Louie et al., 2017), the parenting role also carries emotional fulfillment, personal meaning, and a sense of competence, not only demands and burdens (for a review, see Nomaguchi & Milkie, 2020). Reflecting this duality, Berry and Jones (1995) proposed that parenting stress comprises both a negative component, capturing parenting burden, and a positive component, reflecting parenting reward. Subsequent validation studies have supported this two-dimensional structure across cultures, including Chinese (Cheung, 2000) and Norwegian (Nærde & Hukkelberg, 2020) samples. These two dimensions are relatively independent. Mothers with similar overall parenting stress scores may differ substantially in the balance between parenting burden and parenting reward (Oyarzún-Farías et al., 2021). Person-centered analyses of separate indicators can reveal such subgroups, which a single composite score would obscure (Horton et al., 2024). This variation may be particularly pronounced under contemporary intensive mothering norms, which place increasing demands on mothers while also shaping the rewards they derive from parenting (Ennis, 2014; Novoa et al., 2025). Most previous studies, however, have relied on a single composite score or variable-centered approaches that emphasize average associations between parenting stress and children’s outcomes. This approach may overlook distinct combinations of positive and negative parenting stress across mothers (Magnusson, 2003). Person-centered approaches, such as latent profile analysis (LPA), address this gap by identifying subgroups of mothers with similar configurations of parenting burden and parenting reward (Shi & Tan, 2021; Wang & Cheng, 2025).

Among the parenting behaviors associated with parenting stress, overparenting is likely to be particularly relevant in contemporary Chinese families. Overparenting is characterized by parental attention, protection, and intervention that exceed children’s developmental needs (Jiao & Segrin, 2022). Because parenting stress comprises both a negative and a positive dimension, these two dimensions may relate to overparenting through different mechanisms. On one hand, self-determination theory suggests that threats to psychological needs elicit compensatory efforts to restore a sense of control (Bartholomew et al., 2011). Mothers experiencing heightened negative strain may therefore become more controlling or intrusive as a way of reducing uncertainty and protecting their children, consistent with evidence that mothers whose psychological needs are frustrated engage more frequently in controlling parenting practices (Slobodin et al., 2020). On the other hand, overparenting may also stem from positive over-investment in the parenting role rather than anxious control (Segrin et al., 2013). Under contemporary intensive mothering norms, which expect mothers to actively support their children’s development while minimizing potential risks (Ennis, 2014), mothers who find parenting rewarding may channel that investment into greater supervision, protection, and involvement. Parenting stress may therefore relate to overparenting through two distinct pathways, one rooted in strain and control restoration, the other in positive engagement with the parenting role.

Whether elevated overparenting under such circumstances benefits or hinders development remains unclear. Evidence for overparenting as maladaptive comes mainly from adolescents and emerging adults in Western individualistic societies, where it can undermine autonomy and increase maladjustment (Cui et al., 2022). Because young children are still developing self-regulatory capacities and rely on parental guidance and emotional support (Kopp, 1989), parental behaviors that would be coded as excessive at older ages may be difficult to distinguish from ordinary involvement in early childhood. Cultural context may further shape how intensive parental involvement is understood. Research on Chinese parenting suggests that parental protection, directiveness, and involvement are culturally salient practices, conceptualized differently from analogous behaviors in Western contexts (Y. Li & Vandenbroeck, 2020; Wu et al., 2002). Parenting behaviors labeled as overparenting in Western contexts may thus function differently in contemporary Chinese families.

In summary, although parenting stress has been consistently linked to young children’s social–emotional competence, three questions remain unresolved. First, whether overparenting functions as a behavioral pathway linking maternal parenting stress to children’s social–emotional competence has rarely been examined, particularly during early childhood and in non-Western cultural contexts. Second, parenting stress has often been treated as a homogeneous and predominantly negative construct, overlooking how its positive and negative dimensions combine across mothers. Third, whether the association between overparenting and children’s social–emotional competence is consistent across mothers, or varies according to their broader parenting stress profiles, has yet to be tested. As fertility continues to decline and each child’s development draws growing attention, answering these questions may help identify families at greater risk and inform more targeted parenting support interventions. To address these gaps, the present study integrated variable-centered and person-centered approaches with three objectives: (1) to examine whether overparenting mediates the association between maternal parenting stress (negative strain and positive experience) and young children’s social–emotional competence; (2) to identify distinct profiles of maternal parenting stress based on its positive and negative dimensions using latent profile analysis; and (3) to test whether the association between overparenting and children’s social–emotional competence differs across these profiles.

Based on this rationale, we proposed three hypotheses. First, we hypothesized that both dimensions of parenting stress would predict overparenting, albeit via different mechanisms: negative strain would positively predict overparenting via a compensatory control pathway (H1a), while positive experience would positively predict overparenting via a positive-investment pathway (H1b); in turn, overparenting was expected to carry significant indirect effects linking each dimension to children’s social–emotional competence. Second, because positive experience and negative strain reflect relatively independent aspects of parenting stress rather than opposite ends of a single dimension (Cheung, 2000; Nærde & Hukkelberg, 2020; Oyarzún-Farías et al., 2021), we hypothesized that latent profile analysis would identify qualitatively distinct maternal subgroups defined by different combinations of these two dimensions, rather than subgroups that vary only in overall stress severity (H2). Third, because contemporary intensive mothering norms place similar demands for involvement on mothers regardless of how much stress that involvement entails (Ennis, 2014; Novoa et al., 2025), we hypothesized that the association between overparenting and children’s social–emotional competence would differ across these profiles, and would be weaker among mothers experiencing higher strain (H3).

## 2. Materials and Methods

### 2.1. Participants

This study employed a cross-sectional, nonexperimental design (Von Elm et al., 2007). Participants were mothers of children enrolled in six kindergartens and first-grade classes of three primary schools in northern Guangdong Province, China. Questionnaires were distributed to eligible mothers using convenience sampling, and 481 were returned. Responses were screened using three criteria. Participants who failed any of three attention-check items (e.g., an instruction to select “strongly disagree” if responding attentively) were excluded (*n* = 21). Response patterns showing no variation across items, indicating careless responding, were also removed (*n* = 2). Completion times exceeding 3 *SD* above the mean (*M* = 545.7 s, *SD* = 456.7 s, i.e., >1916 s) were excluded as atypically long (*n* = 13). These criteria excluded 36 questionnaires, yielding 445 valid responses (effective response rate = 92.52%). No missing data were present in the final sample. Informed consent was obtained from all participants.

### 2.2. Measures

#### 2.2.1. Parenting Stress

Maternal parenting stress was measured with the 18-item Parental Stress Scale (PSS; Berry & Jones, 1995), which has been validated in Chinese samples (Cheung, 2000). Items are rated on a 5-point Likert scale (1 = strongly disagree, 5 = strongly agree). Eight positively worded items (1, 2, 5, 6, 7, 8, 17, and 18) were reverse scored. Two dimensions were created based on the original factor structure (Berry & Jones, 1995): positive experience (e.g., “I am happy in my role as a parent”, α = 0.81) and negative strain (e.g., “I feel overwhelmed by the responsibility of being a parent”, α = 0.89). A confirmatory factor analysis of this two-factor structure showed fit below conventional thresholds, χ^2^/*df* = 7.97, CFI = 0.802, TLI = 0.770, RMSEA = 0.125, SRMR = 0.173, though it fit significantly better than a one-factor alternative, Δχ^2^ = 794.72, *df* = 1, *p* < 0.001. The low VIFs (1.02–1.29) indicate that positive experience and negative strain share minimal linear redundancy, ruling out the possibility that they represent a single bipolar construct measured in opposite directions.

#### 2.2.2. Overparenting

Overparenting was measured with the 13-item Locke Parenting Scale (LPS; Locke et al., 2016; overall α = 0.84), which comprises four dimensions: over-happiness (e.g., “I try hard to make sure my child is always happy”, α = 0.90), over-friending (e.g., “I would consider my child to be like a friend now”, α = 0.88), over-guilt (e.g., “I have used guilt to change my child’s behaviour”, α = 0.84), and over-worry (e.g., “I worry about what others think of my parenting”, α = 0.81). Items are rated on a 5-point Likert scale (1 = strongly disagree, 5 = strongly agree), with higher scores indicating higher levels of overparenting. The LPS was translated into Chinese following standard back-translation procedures. Two bilingual researchers independently translated the original items into Chinese and back-translated the Chinese version into English. Discrepancies were resolved through discussion until consensus was reached. The translated items were then reviewed by an expert in developmental psychology for content clarity and cultural appropriateness, and minor wording revisions were made based on their feedback. A confirmatory factor analysis supported the four-factor structure of the LPS in the present sample, χ^2^/*df* = 3.79, CFI = 0.951, TLI = 0.935, RMSEA = 0.079, SRMR = 0.050.

#### 2.2.3. Children’s Social–Emotional Competence

Children’s social–emotional competence was measured with the Chinese version (Hua & Zhou, 2012) of the Preschool Behavioral and Emotional Rating Scale (PreBERS; Nordness et al., 2009). It was completed by mothers reporting on their child’s behavior. The scale comprises 34 items across four dimensions: emotional regulation (11 items; e.g., “can control anger toward others”), school readiness (11 items; e.g., “has language comprehension comparable to peers”), social confidence (6 items; e.g., “is confident”), and family involvement (6 items; e.g., “has a sense of belonging to the family”). Items are rated on a 4-point scale (1 = not at all like the child, 4 = very much like the child), with higher scores indicating higher social–emotional competence. Cronbach’s α for the total scale was 0.97, and subscale reliabilities were likewise good to excellent (emotional regulation: α = 0.94; school readiness: α = 0.94; social confidence: α = 0.89; family involvement: α = 0.91).

#### 2.2.4. Demographic Variables

Mothers completed a demographic questionnaire reporting on their child’s age, child’s sex, only-child status, and their own educational level. Child age was reported in four categories (3–4, 4–5, 5–6 and 6–7 years). Child sex was coded as 0 = female, 1 = male. Only-child status was assessed by asking whether the target child was an only child (0 = no, 1 = yes). Maternal educational level was reported in four categories: high school or below, associate degree, bachelor’s degree, and graduate degree. Mothers were instructed to complete all child-related measures with reference to their child aged 3–7 years; mothers with more than one child in this age range were asked to report on the oldest child within this range. Child age, child sex, only-child status and maternal educational level were included as covariates in the mediation analysis.

### 2.3. Data Analysis

Data were analyzed using R (version 4.5.2) and SPSS 25.0 (IBM Corp., Armonk, NY, USA). Because kindergarten and first-grade classes differed systematically in child age, we examined clustering across classes within the same grade level prior to the main analyses; intra-class correlations were small (ICCs = 0.01–0.04), indicating no meaningful between-school differences beyond the expected grade-level variation. Harman’s single-factor test was used to assess common method bias. The largest factor accounted for 23.08% of the variance, below the commonly cited critical threshold of 40–50%, suggesting that a single factor does not account for the majority of variance among the study variables (Podsakoff et al., 2003).

Descriptive statistics and bivariate correlations were calculated. Pearson correlations were used for continuous variables, point-biserial correlations for binary variables, and Spearman correlations for ordinal variables. Statistical significance was evaluated at *p* < 0.05 (two-tailed). A sensitivity power analysis indicated that the sample size (*N* = 445) provided 80% power to detect correlations of *r* = 0.13 and regression effects of *f^2^* = 0.03 (Faul et al., 2009). Correlation strength was interpreted using Cohen’s (1988) conventions, with absolute correlation coefficients (|*r*|) below 0.10, 0.10–0.29, 0.30–0.49, and ≥0.50 interpreted as negligible, small, moderate, and large, respectively.

The main analyses combined variable-centered and person-centered approaches. First, a mediation model was estimated with positive experience and negative strain as simultaneous predictors, overparenting as the mediator, and children’s social–emotional competence as the outcome, controlling for child age, sex, only-child status, and maternal education. Indirect effects were tested using bias-corrected bootstrap confidence intervals based on 5000 resamples.

Second, a latent profile analysis (LPA) was conducted using the tidyLPA package (version 2.0.2) to identify profiles of maternal parenting stress based on standardized positive experience and negative strain scores. Models were estimated under an equal-variance, zero-covariance specification (Model 1 in the tidyLPA taxonomy, equivalent to mclust’s “EEI” model version 6.1.2), with 3k + 1 free parameters for k profiles (10 for the retained three-profile solution). Model estimation used mclust’s default hierarchical model-based clustering for EM initialization; to assess sensitivity to starting values, the chosen model was re-estimated from 50 random starting classifications, all of which converged to the same maximum log-likelihood as the original solution. Models with one to five profiles were compared using AIC, BIC, SABIC, entropy, the bootstrap likelihood ratio test (BLRT), and profile interpretability and size. Differences between profiles in overparenting and children’s social–emotional competence were examined using Welch’s ANOVA, followed by Games–Howell post hoc comparisons. Eta-squared was reported as the effect size.

Finally, a multi-group path analysis was conducted in lavaan (version 0.6.21) to test whether the association between overparenting and children’s social–emotional competence differed across parenting stress profiles. Robust maximum likelihood (MLR) estimation was used because of non-normality. Profile differences in the overparenting–competence association were tested by comparing models with freely estimated and constrained slopes. A sensitivity analysis excluding the smallest profile was also conducted. Because most-likely-profile assignment does not account for classification uncertainty, the moderation analysis was additionally repeated using the BCH approach (Bolck et al., 2004).

## 3. Results

### 3.1. Descriptive Statistics and Correlation Analyses

Sample characteristics are presented in Table 1. The sample included 220 boys (49.4%) and 225 girls (50.6%). Regarding age, 93 children (20.9%) were younger than 4 years, 90 (20.2%) were 4–5 years old, 149 (33.5%) were 5–6 years old, and 113 (25.4%) were 6–7 years old (*M* = 5.13 years, *SD* = 1.08); 57.3% of the children were only children. With respect to maternal education, 26.4% of mothers had completed senior high school or secondary vocational school, 35.9% held an associate degree, 36.6% held a bachelor’s degree, and 1.1% held a postgraduate degree.

Descriptive statistics and bivariate correlations among study variables and covariates are presented in Table 2. The two parenting stress dimensions were moderately negatively correlated with each other (*r* = −0.30, *p* < 0.001), supporting their treatment as distinct components rather than a single construct. Positive experience was weakly positively correlated with overparenting (*r* = 0.27, *p* < 0.001), and was positively correlated, with moderate-to-large effects, with all four dimensions of social–emotional competence as well as the total score (*r*s = 0.46–0.58, all *p* < 0.001). In contrast, negative strain showed negligible, non-significant correlations with overparenting (*r* = −0.06) and with each dimension of social–emotional competence (*r*s = 0.01–0.07, all ns). Overparenting was positively correlated, with small-to-moderate effects, with all dimensions of social–emotional competence and the total score (*r*s = 0.26–0.30, all *p* < 0.001).

Several covariates were also associated with the study variables, generally with small-to-moderate effects. Only-child status was correlated with positive experience (*r* = −0.28), negative strain (*r* = 0.30), and overparenting (*r* = −0.25, all *p* < 0.001). Child age was correlated with negative strain (*r* = 0.32, *p* < 0.001) and overparenting (*r* = −0.15, *p* < 0.01). Maternal education showed a small but significant correlation with overparenting (*r* = 0.16, *p* < 0.001), but was not significantly correlated with either parenting stress dimension or social–emotional competence. Child gender was not significantly correlated with any study variable except negative strain, with which it showed a significant but negligible correlation (*r* = 0.09, *p* < 0.05).

### 3.2. Variable-Centered Analyses

**Figure 1:**
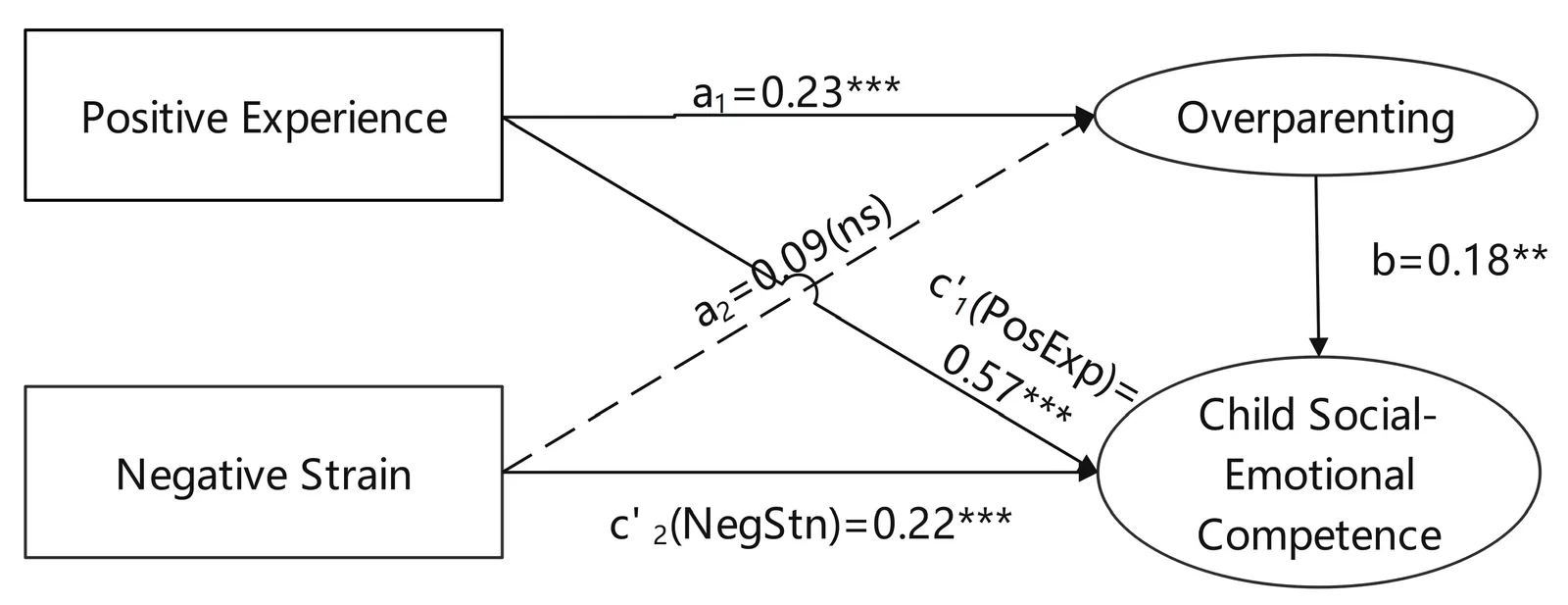
Variable-centered mediation model with positive experience and negative strain entered simultaneously as predictors, overparenting as the mediator, and children’s social–emotional competence as the outcome, controlling for child age, sex, only-child status, and maternal education. Values are standardized coefficients; *a*_1_ and *a*_2_ represent the effects of each predictor on overparenting, *b* represents the effect of overparenting on social–emotional competence, and the paths labeled *c′* represent the direct effect of each predictor on social–emotional competence after accounting for overparenting. Solid lines indicate significant paths; the dashed line indicates a non-significant path (ns = not significant). PosExp = positive experience; NegStn = negative strain. ** *p* < 0.01. *** *p* < 0.001.

A mediation model was estimated with positive experience and negative strain entered simultaneously as predictors, overparenting as the mediator, and children’s social–emotional competence as the outcome, controlling for child age, sex, only-child status, and maternal education (Figure 1). Positive experience significantly and positively predicted overparenting (*a*_1_ = 0.23, *p* < 0.001), whereas negative strain did not significantly predict overparenting (*a*_2_ = 0.09, *p* = 0.111). Overparenting, in turn, positively predicted social–emotional competence (*b* = 0.18, *p* = 0.001). The indirect effect of positive experience through overparenting was significant (indirect effect = 0.04, 95% CI [0.01, 0.07], *p* = 0.010). In contrast, the indirect effect of negative strain through overparenting was not significant (indirect effect = 0.02, 95% CI [−0.01, 0.04], *p* = 0.157). The direct effects of both positive experience (*c′*_1_ = 0.57, *p* < 0.001) and negative strain (*c′*_2_ = 0.22, *p* < 0.001) on social–emotional competence remained significant, and the corresponding total effects were also significant (total effect, positive experience = 0.61, *p* < 0.001; total effect, negative strain = 0.24, *p* < 0.001, see Table 3). Among covariates, only-child status significantly predicted overparenting (β = −0.21, *p* < 0.001), and maternal education significantly predicted overparenting (β = 0.15, *p* < 0.001), but neither predicted social–emotional competence. These results show that the pattern observed in the unadjusted model held after accounting for child age, sex, only-child status, and maternal education.

### 3.3. Latent Profile Analysis of Maternal Parenting Stress

Using the two parenting stress dimensions (positive experience and negative strain) as indicators, latent profile models with one to five profiles were estimated successively. Model fit results are presented in Table 4. As the number of profiles increased, AIC and SABIC decreased, and the BLRT was significant (*p* < 0.05) for every solutions. BIC decreased substantially from the two- to three-profile solution, increased slightly for the four-profile solution, and decreased again for the five-profile solution. Thus, no single information criterion unambiguously identified an optimal number of profiles within this range. Entropy, by contrast, was highest for the three-profile model (0.836) and declined for the four- and five-profile models, indicating that the three-profile solution provided the clearest classification. Examination of the four-profile solution showed that the additional profile did not represent a qualitatively distinct combination of positive experience and negative strain and did not provide gains in interpretability. Balancing fit indices, entropy, profile size, and theoretical interpretability, the three-profile model was retained as the optimal solution.

Classification diagnostics further supported the three-profile solution. Average posterior probabilities of assigned-class membership were 0.98, 0.93, and 0.91 for the disengaged, engaged–high-strain, and engaged–low-strain profiles, respectively, indicating high classification precision across all three profiles, including the smallest (*n* = 17).

**Figure 2:**
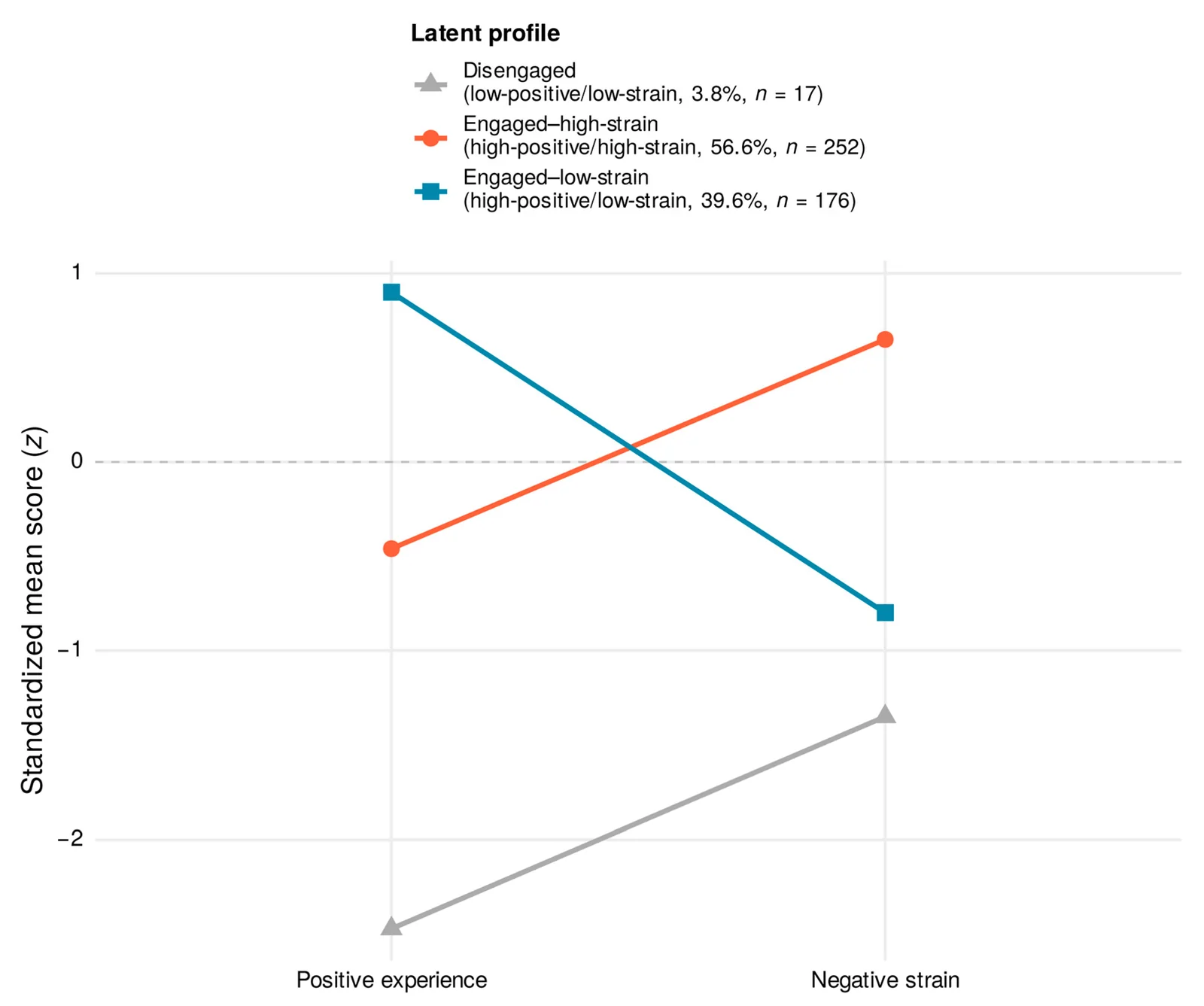
Standardized mean scores (z scores; *M* = 0, *SD* = 1) on the two parenting stress dimensions (positive experience and negative strain) for the three latent profiles. Profile 1 = disengaged (low-positive/low-strain, *n* = 17); Profile 2 = engaged–high-strain (high-positive/high-strain, *n* = 252); Profile 3 = engaged–low-strain (high-positive/low-strain, *n* = 176).

The three profiles were named according to their characteristics on the two parenting stress dimensions (Figure 2). Profile 1 (3.8%, *n* = 17) scored low on both positive experience and negative strain, indicating limited emotional engagement in parenting together with a low perceived burden; it was labeled the disengaged profile. Profile 2 (56.6%, *n* = 252), the largest group, scored moderately high on positive experience but high on negative strain, reflecting substantial parenting involvement accompanied by considerable stress and loss of control; it was labeled the engaged–high-strain profile. Profile 3 (39.6%, *n* = 176) scored high on positive experience but low on negative strain, indicating strong engagement and emotional gratification with little subjective strain; it was labeled the engaged–low-strain profile.

### 3.4. Class Differences in Overparenting and Children’s Social–Emotional Competence

The profiles differed significantly on all overparenting dimensions and all dimensions of children’s social–emotional competence (Table 5), with effect sizes (η^2^) ranging from small (over-guilt, η^2^ = 0.036) to large (family involvement, η^2^ = 0.341).

For children’s social–emotional competence, a consistent ordering emerged across all four dimensions (emotional regulation, school readiness, social confidence, family involvement), with the engaged–low-strain profile scoring highest, the engaged–high-strain profile intermediate, and the disengaged profile lowest (all pairwise comparisons significant, *p*s < 0.001).

The profiles also differed in their patterns of overparenting, although the differences varied across subdimensions. The engaged–low-strain profile scored highest on over-happiness and over-friending. The difference from the engaged–high-strain profile was significant for over-happiness but not for over-friending. The disengaged profile scored highest on over-guilt, differing significantly from both the engaged–high-strain (*p* = 0.006) and engaged–low-strain (*p* = 0.002) profiles. It also scored higher on over-worry than the engaged–low-strain profile (*p* = 0.003), but not the engaged–high-strain profile (*p* = 0.086). For overall overparenting, the disengaged profile differed significantly from the engaged–low-strain profile (*p* = 0.005), but not from the engaged–high-strain profile (*p* = 0.166).

### 3.5. Moderating Role of Parenting Stress Profiles in the Relation Between Overparenting and Social–Emotional Competence

To test whether parenting stress profiles moderated the association between overparenting and children’s social–emotional competence, a multi-group path analysis using observed composite scores was conducted in lavaan. Social–emotional competence was regressed on overparenting separately within each profile. The comparison was significant, Δχ^2^(2) = 12.37, *p* = 0.002, indicating that the association differed across the three profiles.

**Figure 3:**
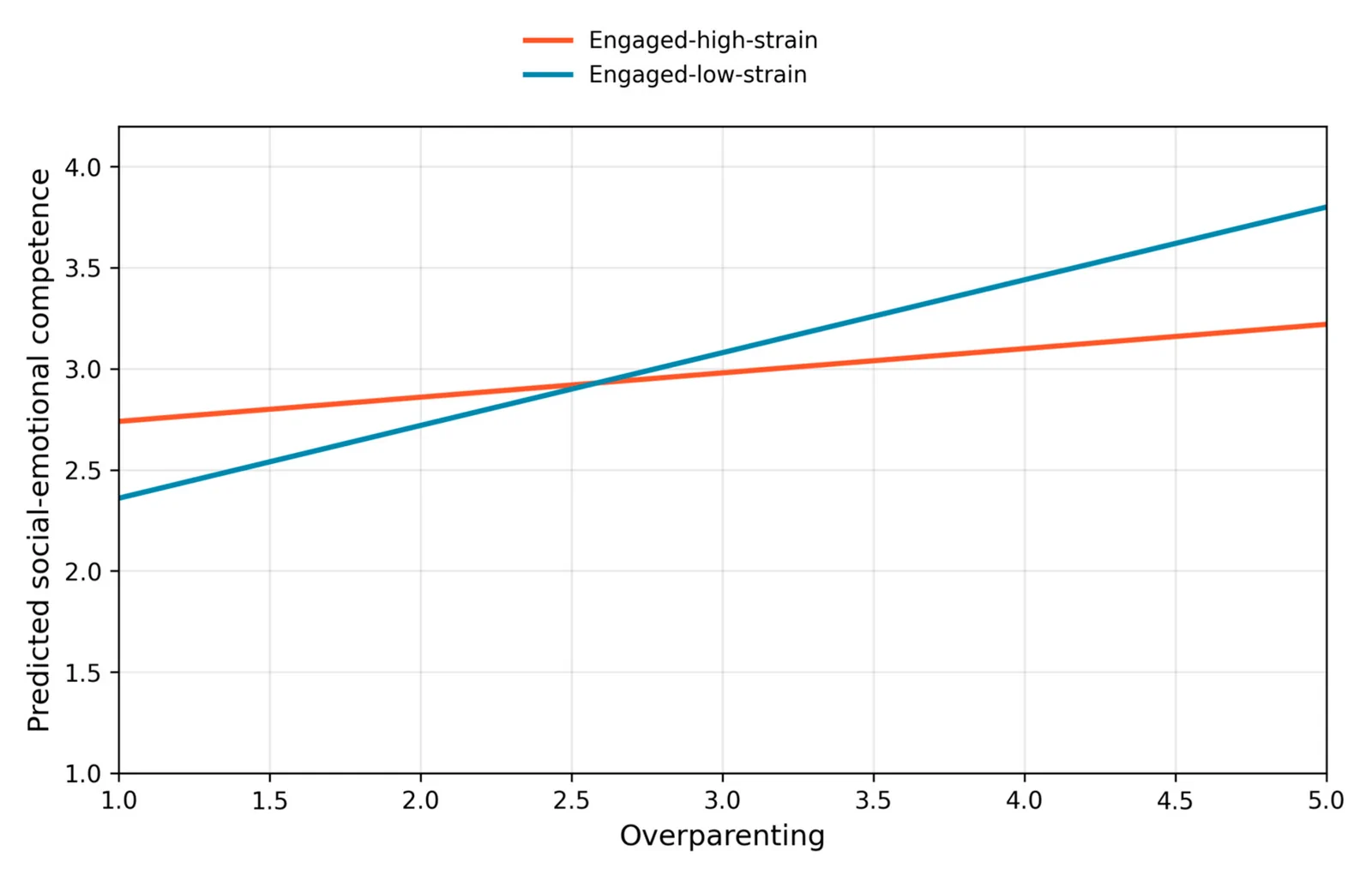
Moderating effect of parenting stress class profile on the relation between overparenting and children’s social–emotional competence. Lines show predicted social–emotional competence based on the within-profile slopes estimated in the multi-group path analysis (Table 6) for the engaged–high-strain (*n* = 252) and engaged–low-strain (*n* = 176) profiles. The disengaged profile (*n* = 17) is not shown because its estimates were unstable (see Appendix A).

Overparenting was positively associated with social–emotional competence in the engaged–high-strain profile, *b* = 0.12, *SE* = 0.06, *p* = 0.036, and the engaged–low-strain profile, *b* = 0.36, *SE* = 0.08, *p* < 0.001, and this association was significantly stronger in the engaged–low-strain profile, *b*_diff_ = −0.24, 95% CI [−0.44, −0.04], *p* = 0.016 (Figure 3). A sensitivity analysis excluding the disengaged profile yielded a significant moderation effect, Δχ^2^(1) = 6.78, *p* = 0.009, supporting the robustness of this difference between the two engaged profiles. Estimates for the disengaged profile (*n* = 17) were unstable across model specifications. The association was non-significant under standard maximum likelihood estimation but significant under robust MLR estimation. Therefore, we do not interpret this profile substantively here.

**Table 6:** Multi-group path analysis of the association between overparenting and children’s social–emotional competence across parenting stress profiles (robust MLR estimation).

Panel A. Within-Profile Slopes (Overparenting → Social–Emotional Competence)				
Profile	*b*	*SE*	*p*	95% CI
Engaged–high-strain (*n* = 252)	0.12	0.06	0.036	[0.01, 0.24]
Engaged–low-strain (*n* = 176)	0.36	0.08	<0.001	[0.20, 0.52]
**Panel B. Tests of Slope Differences**				
**Comparison**		**Δχ^2^ (Scaled)**	***df***	***p***
Overall moderation (free vs. equal slopes)		12.37	2	0.002
Engaged–high-strain vs. engaged–low-strain		*z* = −2.40	1	0.016
Sensitivity: two engaged profiles only (excluding disengaged)		6.78	1	0.009

Note. *b* = unstandardized within-profile regression slope; *SE* = standard error; CI = confidence interval; MLR = robust maximum likelihood; Δχ^2^ (scaled) = scaled chi-square difference test comparing models with freely estimated versus equality-constrained slopes; *df* = degrees of freedom; *z* = Wald test of the difference between the two slopes. Overparenting was mean-centered. Estimation used robust maximum likelihood, providing standard errors and scaled test statistics robust to non-normality. The disengaged profile (*n* = 17) is not reported here (see Appendix A.1 and Appendix A.2). The overall moderation test (*df* = 2) includes all three profiles; the sensitivity analysis (*df* = 1) confirms the finding holds when the disengaged profile is excluded entirely.

## 4. Discussion

The present study examined how mothers’ positive parenting experience and negative parenting strain relate to overparenting and young children’s social–emotional competence, using both variable-centered and person-centered approaches. Together, the two approaches point to a common conclusion: parenting stress is not a uniformly detrimental construct, and its association with children’s development depends on which component of stress is considered and on how positive experience and strain co-occur within individual mothers. As discussed below, this distinction has both theoretical and practical implications.

Parenting stress is not functionally unidimensional. The latent profile analysis identified three profiles of maternal parenting stress. A disengaged profile showed low positive experience and low strain. An engaged–high-strain profile combined moderate positive experience with high stress and loss of control. An engaged–low-strain profile showed high positive experience and low strain. The latter two profiles were similarly high in parental involvement but differed markedly in stress and loss of control. This structure differs from previous latent profile research on Chinese parents (Wang & Cheng, 2025). Using the PSI-SF, Wang and Cheng (2025) identified four profiles ordered primarily along a single dimension of stress severity. The present two-dimensional approach instead revealed mothers who were similarly engaged in parenting but differed in whether that engagement came with high or low strain. The predominance of the engaged–high-strain profile reflects the realities of intensive mothering norms in contemporary urban China, under which mothers highly invested in child-rearing may also carry substantial stress and responsibility (Ennis, 2014; Novoa et al., 2025). Although the two studies used different measures and profile structures, both identified a relatively large group characterized by elevated parenting stress, suggesting that high parenting stress may be common among Chinese parents of young children (M. Li et al., 2017). However, intensive parenting behaviors cannot be classified as universally adaptive or maladaptive. In early childhood, intensive involvement functions as a positive correlate of child competence.

Family context may help explain why mothers experience different combinations of parenting reward and strain even when their total stress levels appear similar (Östberg & Hagekull, 2000). For example, Chinese parents in two-child families report higher parenting stress, lower social support, and lower parenting self-efficacy than parents in one-child families (Hong & Liu, 2019). Such evidence suggests that maternal parenting stress appears to depend not only on the intensity of parenting demands but also on the resources available to meet them. The present latent profile analysis builds on this by showing that how positive experience and strain combine, not the total stress score, is what distinguishes mothers in ways relevant to children’s social–emotional competence.

The variable-centered results add an important qualification. Only positive experience, not negative strain, significantly predicted overparenting and carried a significant indirect effect on children’s social–emotional competence. This runs counter to the assumption, drawn from self-determination theory, that overparenting mainly emerges as a compensatory response to parenting strain. Instead, mothers who found parenting rewarding appeared more likely to become highly involved, consistent with the idea that overparenting can stem from positive over-investment in the parenting role rather than anxious control (Segrin et al., 2013). This does not rule out a role for strain. Both positive experience and negative strain retained significant direct effects on social–emotional competence after accounting for overparenting, indicating that strain still shapes children’s outcomes through pathways beyond overparenting itself. Among covariates, mothers of only children reported less overparenting, which may reflect fewer competing demands on parental time and attention when siblings are absent. Maternal education positively predicted overparenting, possibly because more educated mothers are more exposed to, and more responsive to, intensive parenting norms that emphasize close monitoring and involvement (Novoa et al., 2025). This interpretation assumes a causal direction that the cross-sectional design cannot confirm (Maxwell & Cole, 2007). The same pattern is consistent with reverse or reciprocal effects. A child who is already well-regulated may draw more rewarding interactions from the mother, rather than her positive experience driving greater involvement. Likewise, a child’s early emotional or behavioral difficulties may increase maternal strain, rather than strain shaping the child’s later competence. Longitudinal or cross-lagged data are needed to distinguish these directions from the one we propose.

More importantly, maternal parenting stress profiles moderated the association between overparenting and young children’s social–emotional competence. This association was positive and strongest among engaged–low-strain mothers, and weaker among engaged–high-strain mothers, despite comparable levels of engagement. This fits the variable-centered finding that positive experience, rather than negative strain, predicted higher overparenting. One possible explanation involves the emotional quality of parent-child interactions. Elevated parenting stress is linked to less emotionally supportive and more tension-laden interactions (Deater-Deckard, 1998; Slobodin et al., 2020). When high involvement occurs alongside substantial strain, children may experience it as less supportive, weakening its association with social–emotional competence. Mothers with high positive experience and low strain, by contrast, may be better able to provide involved parenting within a warm emotional climate (Zhang et al., 2019). Overparenting therefore does not appear to be a uniform risk factor in the early years. Its developmental correlates depend on the emotional context in which it occurs.

This pattern differs from findings among adolescents and emerging adults, where higher overparenting has more often been linked to poorer autonomy and adjustment (Cui et al., 2022; Schoppe-Sullivan et al., 2024). Because the LPS was developed for school-aged children, some of its subscales may not translate cleanly to this age group. The subscales of over-happiness and over-friending concern a mother’s involvement in her child’s mood and peer relationships and may capture ordinary warmth and responsiveness in a 4- to 7-year-old, whereas over-guilt and over-worry more directly index anxious, control-driven behavior. The positive association observed here may therefore partly reflect beneficial engagement rather than the intrusive, autonomy-limiting involvement the construct was designed to capture. Future work should test with preschool-validated measures that separate supportive involvement from developmentally excessive intervention. Notably, overparenting did not become harmful in the engaged–high-strain profile, where tense mother–child interactions might be expected to turn intrusive involvement into a liability. Across all three profiles, its association with social–emotional competence remained positive or null, never negative. This pattern is inconsistent with overparenting functioning as intrusive over-control, which should be most detrimental precisely when parental strain is high. It instead supports the interpretation above that the LPS composite used here likely captures parental involvement more broadly rather than the anxious, controlling behavior the construct was designed to index.

The present study extends previous research in two ways. First, it clarifies the associations between overparenting, maternal parenting experiences, and children’s social–emotional competence. Second, it shows that these associations vary across maternal parenting-stress profiles rather than being uniform across mothers. These findings suggest that interventions may benefit from considering mothers’ broader patterns of parenting stress rather than focusing solely on overall stress levels or parenting involvement. The predominance of the engaged–high-strain profile indicates that many Chinese mothers remain highly involved in child-rearing despite experiencing substantial strain. For these mothers, increasing parental involvement may have limited additional value because their involvement is already high. Instead, support aimed at reducing the burden associated with intensive parenting may be more relevant. Such support could include strategies to reduce parenting-related burnout and stress, strengthen social support, and facilitate a more balanced distribution of caregiving responsibilities. These approaches may help mothers sustain their involvement without the potential costs associated with persistent strain. For mothers in the disengaged profile, a different set of needs may be relevant. Although strain was relatively low in this group, their lower levels of engagement suggest that support focused on strengthening parenting self-efficacy, positive emotional connection, and the rewarding aspects of the parenting role may be useful. Attention to potential feelings of anxiety or guilt may also be warranted. Overall, these findings highlight the potential value of tailoring support to different patterns of maternal parenting stress and engagement, with the broader goal of promoting maternal well-being and children’s social–emotional competence.

Several limitations should be acknowledged. First, the cross-sectional design precludes causal inference; longitudinal research is needed to examine the stability of parenting stress profiles and their long-term predictive effects. Second, all constructs were assessed via maternal self-report within the same assessment, raising the possibility of shared method variance. Although Harman’s single-factor test did not indicate an extreme common-factor solution, this test provides only a preliminary indication and cannot fully rule out common method bias. Shared-rater effects, social desirability, and general response tendencies therefore remain plausible explanations for part of the observed associations. Third, the sample was drawn through convenience sampling from a single region in northern Guangdong Province, which may limit the generalizability of the findings to other populations. Fourth, apart from child age, child sex, only-child status, and maternal education, other sociodemographic factors such as marital status, employment status, and family income were not assessed and could not be controlled for. Fifth, the disengaged profile comprised only 17 mothers, which limited the precision and stability of estimates involving this group; the association between overparenting and social–emotional competence in this profile was therefore not interpreted substantively. Future research with larger and more demographically balanced samples is needed to examine these associations more reliably. Finally, the overparenting measure was originally developed for parents of school-aged children, and validated measures for parents of preschool-aged children remain limited, particularly in Chinese samples. Although its four-factor structure was supported in the present sample, some dimensions, such as over-happiness and over-friending, may partly reflect developmentally appropriate parental warmth in early years, and its Chinese translation was reviewed by only one content expert, which limits the strength of its content validity evidence.

## 5. Conclusions

Positive experience, but not negative strain, was indirectly associated with children’s social–emotional competence through overparenting, whereas negative strain showed a direct but not indirect association. Maternal parenting stress was not a homogeneous construct. Distinct profiles characterized by different combinations of positive parenting experience and parenting strain were identified, and these profiles differed in children’s social–emotional competence. The association between overparenting and children’s social–emotional competence also varied across maternal parenting stress profiles and was significantly stronger among mothers reporting high positive experience and low strain than among mothers experiencing high strain. These findings suggest that overparenting was not uniformly associated with children’s social–emotional competence during the early years. Instead, its association may be better understood in the context of the heterogeneous nature of maternal parenting stress.

## Figures and Tables

**Table 1:** Sample characteristics (*N* = 445).

Variable	Category	*n*	%
Child age	6–7 years	113	25.4
	5–6 years	149	33.5
	4–5 years	90	20.2
	3–4 years	93	20.9
Child sex	Male	220	49.4
	Female	225	50.6
Only-child status	No	190	42.7
	Yes	255	57.3
Maternal education	High school or below	116	26.4
	Associate degree	158	35.9
	Bachelor’s degree	161	36.6
	Graduate degree	5	1.1

Note. *N* = 445. *n* = number of participants. Data on maternal education were missing for 5 mothers; percentages for this variable are based on valid responses (*n* = 440).

**Table 2:** Descriptive statistics and correlations among covariates and study variables (*N* = 445).

	*M*	*SD*	1	2	3	4	5	6	7	8	9	10	11
1. Child age	—	—	—										
2. Child sex	—	—	−0.06	—									
3. Only-child status	—	—	0.34 ***	0.04	—								
4. Maternal education	—	—	0.05	0.03	0.10 *	—							
5. Positive experience	3.97	0.57	−0.20 ***	−0.07	−0.28 ***	0.02	—						
6. Negative strain	3.35	0.96	0.32 ***	0.09 *	0.30 ***	0.00	−0.30 ***	—					
7. Overparenting	3.45	0.68	−0.15 **	−0.08	−0.25 ***	0.16 ***	0.27 ***	−0.06	—				
8. Emotional regulation	2.98	0.68	−0.07	0.04	−0.07	0.01	0.47 ***	0.05	0.26 ***	—			
9. School readiness	3.08	0.67	−0.12 *	0.04	−0.07	0.04	0.46 ***	0.04	0.28 ***	0.89 ***	—		
10. Social confidence	3.17	0.65	−0.12 *	0.07	−0.11 *	0.02	0.47 ***	0.01	0.27 ***	0.61 ***	0.61 ***	—	
11. Family involvement	3.40	0.64	−0.12 *	0.03	−0.08	0.07	0.58 ***	0.07	0.28 ***	0.68 ***	0.68 ***	0.67 ***	—
12. SEC (total)	3.12	0.59	−0.13 **	0.05	−0.09	0.03	0.54 ***	0.05	0.30 ***	0.94 ***	0.94 ***	0.77 ***	0.82 ***

Note. *M* = mean; *SD* = standard deviation; SEC = social–emotional competence. Child sex was coded 0 = female, 1 = male; only-child status was coded 0 = only child, 1 = non-only child. SEC = social–emotional competence. *M/SD* are not reported for child age and maternal education, as these are ordinal variables; see Table 1 for their frequency distributions. Correlations involving child age or maternal education were computed using Spearman’s ρ; all other correlations use Pearson’s r (point-biserial for binary variables). * *p* < 0.05, ** *p* < 0.01, *** *p* < 0.001.

**Table 3:** Results of variable-centered mediation analyses (covariate-adjusted).

Path	β	*SE*	*p*	95% CI
Positive experience → Overparenting (*a*_1_)	0.23	0.05	<0.001	[0.13, 0.32]
Negative strain → Overparenting (*a*_2_)	0.09	0.06	0.111	[−0.02, 0.21]
Overparenting → SEC (*b*)	0.18	0.06	0.001	[0.07, 0.28]
Positive experience → SEC (*c’*_1_, direct)	0.57	0.04	<0.001	[0.50, 0.64]
Negative strain → SEC (*c’*_2_, direct)	0.22	0.05	<0.001	[0.12, 0.32]
Indirect effect, positive experience (*a*_1_ × *b*)	0.04	0.02	0.010	[0.01, 0.07]
Indirect effect, negative strain (*a*_2_ × *b*)	0.02	0.01	0.157	[−0.01, 0.04]
Total effect, positive experience	0.61	0.03	<0.001	[0.54, 0.67]
Total effect, negative strain	0.24	0.05	<0.001	[0.14, 0.34]

Note. Values are standardized coefficients (β). SE = standard error; CI = confidence interval; SEC = social–emotional competence. *a*_1_ and *a*_2_ = effects of positive experience and negative strain on overparenting; *b* = effect of overparenting on SEC; *c’*_1_ and *c’*_2_ = direct effects of positive experience and negative strain on SEC, controlling for overparenting. Model controls for child age, sex, only-child status, and maternal education (see text for covariate effects). The confidence interval for the indirect effect was obtained using bias-corrected bootstrapping with 5000 resamples.

**Table 4:** Fit indices for the latent profile models.

Profiles	LogLik	AIC	BIC	SABIC	Entropy	BLRT	BLRT (*p*)	Profile Proportions
1	−1261.85	2531.71	2548.10	2535.41	—	—	—	1.00 (445)
2	−1221.83	2457.66	2486.35	2464.13	0.679	80.05	0.010	0.50/0.50 (223/222)
**3**	**−1152.95**	**2325.91**	**2366.89**	**2335.15**	**0.836**	**137.75**	**0.010**	**0.04/0.57/0.40** **(17/252/176)**
4	−1144.06	2314.11	2367.39	2326.13	0.779	17.80	0.020	0.04/0.43/0.37/0.16 (18/193/169/65)
5	−1127.51	2287.03	2352.60	2301.82	0.780	33.08	0.010	0.04/0.35/0.26/0.18/0.17 (16/156/120/81/72)

Note. LogLik = log-likelihood; AIC = Akaike information criterion; BIC = Bayesian information criterion; SABIC = sample-size-adjusted BIC; BLRT = bootstrap likelihood ratio test comparing the k − 1 vs. k profile solution, based on 100 bootstrap draws. k = number of profiles. Profile proportions are followed by the number of mothers in each profile in parentheses. The retained three-profile solution is shown in bold. *N* = 445.

**Table 5:** Differences among maternal parenting stress profiles in overparenting and children’s social–emotional competence.

Variable	P1 *M (SE)*	P2 *M (SE)*	P3 *M (SE)*	Welch’s *F*(2, *df*)	η^2^	Post hoc (Games-Howell)
Over-happiness	2.19 (0.21)	4.10 (0.05)	4.42 (0.05)	58.90 (2, 43.0) ***	0.264	P1 < P2, P1 < P3, P2 < P3
Over-friending	3.78 (0.24)	3.18 (0.08)	4.20 (0.04)	59.71 (2, 43.1) ***	0.179	P2 < P3
Over-guilt	3.67 (0.25)	2.75 (0.07)	2.59 (0.07)	8.64 (2, 44.1) ***	0.036	P1 > P2, P1 > P3
Over-worry	3.35 (0.27)	2.71 (0.07)	2.24 (0.07)	15.50 (2, 43.8) ***	0.062	P1 > P3, P2 > P3
Overparenting (total)	3.08 (0.14)	3.36 (0.05)	3.61 (0.04)	12.66 (2, 44.5) ***	0.045	P1 < P3
Emotional regulation	1.59 (0.10)	2.90 (0.04)	3.24 (0.04)	130.86 (2, 47.3) ***	0.228	P1 < P2, P1 < P3, P2 < P3
School readiness	1.95 (0.12)	2.97 (0.04)	3.34 (0.04)	73.47 (2, 45.6) ***	0.185	P1 < P2, P1 < P3, P2 < P3
Social confidence	2.22 (0.14)	3.06 (0.04)	3.43 (0.04)	49.37 (2, 44.2) ***	0.162	P1 < P2, P1 < P3, P2 < P3
Family involvement	1.72 (0.15)	3.32 (0.03)	3.67 (0.04)	89.32 (2, 43.1) ***	0.341	P1 < P2, P1 < P3, P2 < P3

Note. P1 = disengaged (*n* = 17); P2 = engaged–high-strain (*n* = 252); P3 = engaged–low-strain (*n* = 176). *M* = mean; *SE* = standard error; η^2^ = eta-squared (effect size). Values in parentheses after Welch’s F are the numerator and denominator degrees of freedom. In the post hoc column, “<“ and “>” indicate significant pairwise differences (*p* < 0.05) based on Games–Howell tests. Because Levene’s tests indicated unequal variances across profiles for most dimensions, Welch’s F is reported as the omnibus test throughout for consistency. *** *p* < 0.001.

## Data Availability

The data supporting the findings of this study are openly available via https://osf.io/xeyha/overview?view_only=b3e68272b8eb4f91b408fa7c62537b02 (accessed on 9 October 2026).

## Appendix A

### Appendix A.1. Stability Diagnostics for the Disengaged Profile (n = 17)

Given the small size of the disengaged profile, we conducted three checks on its overparenting–social–emotional competence (OP–SEC) slope estimate (*b* = 0.320, *p* = 0.140).

Jackknife. Refitting the slope with each mother iteratively omitted produced estimates ranging from 0.237 to 0.374 (*SD* = 0.031), with no sign reversals, indicating the point estimate was not driven by individual cases.

Bootstrap CI width. The bootstrap 95% CI for the disengaged profile [−0.01, 0.99] was 3–4 times wider than for the engaged–high-strain [0.01, 0.24] and engaged–low-strain [0.22, 0.54] profiles, reflecting limited power rather than an unstable estimate.

Pairwise comparisons. Bias-corrected bootstrap comparisons showed no significant difference between the disengaged profile and either other profile (vs. engaged–high-strain: Δ = 0.20, 95% CI [−0.15, 0.83], *p* = 0.360; vs. engaged–low-strain: Δ = −0.04, 95% CI [−0.42, 0.56], *p* = 0.851). Only the engaged–high-strain vs. engaged–low-strain comparison was significant (Δ = −0.24, 95% CI [−0.46, −0.06], *p* = 0.018).

### Appendix A.2. Classification-Error-Corrected (BCH) Analysis

To address possible bias from treating profile assignment as observed rather than probabilistic, we repeated the moderation analysis using the BCH method (Bolck et al., 2004), which weights each mother’s contribution to every profile-specific regression by classification-error-corrected weights derived from the posterior classification probability matrix (Table A1), rather than her single most likely assignment. Because these weights can be negative, slopes were estimated via weighted least squares and standard errors via bootstrap (2000 resamples).

**Table A1:** Classification probability matrix.

Assigned Profile	*P* (True = Disengaged)	*P* (True = Engaged-High-Strain)	*P* (True = Engaged-Low-Strain)
Disengaged	0.977	0.021	0.002
Engaged-high-strain	0.009	0.932	0.059
Engaged-low-strain	0.0001	0.089	0.911

Note. Values are average posterior probabilities of membership in each profile (columns) for mothers assigned to each profile (rows); diagonal values indicate classification accuracy.

The central finding replicated: the engaged–low-strain slope (*b* = 0.39, *p* < 0.001) remained significantly stronger than the engaged–high-strain slope (*b* = 0.10, *p* = 0.088), and their difference remained significant (Δ = −0.29, 95% CI [−0.54, −0.04], *p* = 0.020), closely matching the classify-analyze result above. For the disengaged profile, the bootstrap SE increased sharply (*SE* > 40, vs. 0.21 under classify-analyze), reflecting near-singular estimation in a subset of resamples given this profile’s small size and the use of negative correction weights; comparisons involving this profile remained non-significant (both *p*s > 0.5).

## References

[B1-ejihpe-16-00165] Abidin R. R. (1990). Introduction to the special issue: The stresses of parenting. Journal of Clinical Child Psychology.

[B2-ejihpe-16-00165] Anthony L., Anthony B., Glanville D., Naiman D., Waanders C., Shaffer S. (2005). The relationships between parenting stress, parenting behavior and preschoolers’ social competence and behavior problems in the classroom. Infant and Child Development.

[B3-ejihpe-16-00165] Bartholomew K. J., Ntoumanis N., Ryan R. M., Bosch J. A., Thøgersen-Ntoumani C. (2011). Self-determination theory and diminished functioning: The role of interpersonal control and psychological need thwarting. Personality and Social Psychology Bulletin.

[B4-ejihpe-16-00165] Berry J. O., Jones W. H. (1995). The parental stress scale: Initial psychometric evidence. Journal of Social and Personal Relationships.

[B5-ejihpe-16-00165] Bolck A., Croon M., Hagenaars J. (2004). Estimating latent structure models with categorical variables: One-step versus three-step estimators. Political Analysis.

[B6-ejihpe-16-00165] Chen M., Lo C. K. M., Chen Q., Chan K. L., Ip P. (2024). Fertility intention in Hong Kong: Declining trend and associated factors. Applied Research in Quality of Life.

[B7-ejihpe-16-00165] Cheung S.-K. (2000). Psychometric properties of the Chinese version of the parental stress scale. Psychologia: An International Journal of Psychology in the Orient.

[B8-ejihpe-16-00165] Cohen J. (1988). Set correlation and contingency tables. Applied Psychological Measurement.

[B9-ejihpe-16-00165] Cui M., Hong P., Jiao C. (2022). Overparenting and emerging adult development: A systematic review. Emerging Adulthood.

[B10-ejihpe-16-00165] Deater-Deckard K. (1998). Parenting stress and child adjustment: Some old hypotheses and new questions. Clinical Psychology: Science and Practice.

[B11-ejihpe-16-00165] Di H., Hao C., Liu Y., Jiao J., Wang D., Li H. (2026). The association between parenting stress and young children’s social–emotional competence: A moderated mediation model. Frontiers in Psychology.

[B12-ejihpe-16-00165] Domitrovich C. E., Durlak J. A., Staley K. C., Weissberg R. P. (2017). Social-emotional competence: An essential factor for promoting positive adjustment and reducing risk in school children. Child Development.

[B13-ejihpe-16-00165] Ennis L. R. (2014). Intensive mothering: The cultural contradictions of modern motherhood.

[B14-ejihpe-16-00165] Fang Y., Luo J., Boele M., Windhorst D., Van Grieken A., Raat H. (2024). Parent, child, and situational factors associated with parenting stress: A systematic review. European Child & Adolescent Psychiatry.

[B15-ejihpe-16-00165] Faul F., Erdfelder E., Buchner A., Lang A. G. (2009). Statistical power analyses using G* Power 3.1: Tests for correlation and regression analyses. Behavior Research Methods.

[B16-ejihpe-16-00165] Hong X., Liu Q. (2019). Parenting stress, social support and parenting self-efficacy in Chinese families: Does the number of children matter?. Early Child Development and Care.

[B17-ejihpe-16-00165] Horton C., Walsh L. J., Rodriguez A., Kaufman V. A. (2024). The diversity of well-being indicators: A latent profile analysis. Frontiers in Psychology.

[B18-ejihpe-16-00165] Hua M., Zhou R. (2012). Revision of preschool behavioral and emotional rating scale in Chinese preschoolers: Validity and reliability. Chinese Journal of Clinical Psychology.

[B19-ejihpe-16-00165] Jiao J., Segrin C. (2022). The development and validation of the overparenting scale—Short form. Emerging Adulthood.

[B20-ejihpe-16-00165] Kopp C. B. (1989). Regulation of distress and negative emotions: A developmental view. Developmental Psychology.

[B21-ejihpe-16-00165] Li M., Zhang Q., Wang L. (2017). Comparative analysis on parents’ parenting stress and social support between China and Japan in the low-fertility era. Studies in Early Childhood Education.

[B22-ejihpe-16-00165] Li Y., Vandenbroeck M. (2020). Conceptualisations of parent involvement in early childhood education in China. Pacific Early Childhood Education Research Association.

[B23-ejihpe-16-00165] Locke J. Y., Kavanagh D. J., Campbell M. A. (2016). Overparenting and homework: The student’s task, but everyone’s responsibility. Journal of Psychologists and Counsellors in Schools.

[B24-ejihpe-16-00165] Louie A. D., Cromer L. D., Berry J. O. (2017). Assessing parenting stress: Review of the use and interpretation of the parental stress scale. The Family Journal.

[B25-ejihpe-16-00165] Magnusson D. (2003). The person approach: Concepts, measurement models, and research strategy. New Directions for Child and Adolescent Development.

[B26-ejihpe-16-00165] Masarik A. S., Conger R. D. (2017). Stress and child development: A review of the family stress model. Current Opinion in Psychology.

[B27-ejihpe-16-00165] Maxwell S. E., Cole D. A. (2007). Bias in cross-sectional analyses of longitudinal mediation. Psychological Methods.

[B28-ejihpe-16-00165] Nærde A., Hukkelberg S. (2020). An examination of validity and reliability of the Parental Stress Scale in a population based sample of Norwegian parents. PLoS ONE.

[B29-ejihpe-16-00165] Nomaguchi K., Milkie M. A. (2020). Parenthood and well-being: A decade in review. Journal of Marriage and Family.

[B30-ejihpe-16-00165] Nordness P. D., Epstein M. H., Synhorst L. (2009). Convergent validity and test-retest reliability of the preschool behavioral and emotional behavior rating scale: Parents as respondents. Early Childhood Services: An Interdisciplinary Journal of Effectiveness.

[B31-ejihpe-16-00165] Novoa C., Cova F., Nazar G., Oliva K., Vergara-Barra P. (2025). Intensive parenting: The risks of overdemanding. Trends in Psychology.

[B32-ejihpe-16-00165] Oyarzún-Farías M. D. L. A., Cova F., Bustos Navarrete C. (2021). Parental stress and satisfaction in parents with pre-school and school age children. Frontiers in Psychology.

[B33-ejihpe-16-00165] Östberg M., Hagekull B. (2000). A structural modeling approach to the understanding of parenting stress. Journal of Clinical Child Psychology.

[B34-ejihpe-16-00165] Podsakoff P. M., MacKenzie S. B., Lee J.-Y., Podsakoff N. P. (2003). Common method biases in behavioral research: A critical review of the literature and recommended remedies. Journal of Applied Psychology.

[B35-ejihpe-16-00165] Rose-Krasnor L., Denham S., Rubin K. H., Bukowski W. M., Laursen B. (2011). Social-emotional competence in early childhood. Handbook of peer interactions, relationships, and groups.

[B36-ejihpe-16-00165] Santelices M.-P., Cortés P. A. (2022). Mentalization and parental stress: How do they predict mother–child interactions?. Children.

[B37-ejihpe-16-00165] Schoppe-Sullivan S. J., Cui M., Calabrese J. R. (2024). Advancing understanding of overparenting and child adjustment: Mechanisms, methodology, context, and development. Journal of Social and Personal Relationships.

[B38-ejihpe-16-00165] Segrin C., Woszidlo A., Givertz M., Montgomery N. (2013). Parent and child traits associated with overparenting. Journal of Social and Clinical Psychology.

[B39-ejihpe-16-00165] Shi M., Tan C. Y. (2021). Parental autonomy-support, parental control, SES, and mathematics achievement: A latent profile analysis. Journal of Research in Childhood Education.

[B40-ejihpe-16-00165] Slobodin O., Cohen R., Arden A., Katz I. (2020). Mothers’ need frustration and controlling parenting: The moderating role of maternal guilt. Journal of Child and Family Studies.

[B41-ejihpe-16-00165] Sourander J., Salo S., Laakso M.-L., Kalland M. (2025). Toddlers’ social-emotional competence and problem behaviours: Associations with parenting stress and social support among finnish first-time parents. Early Child Development and Care.

[B42-ejihpe-16-00165] Von Elm E., Altman D. G., Egger M., Pocock S. J., Gøtzsche P. C., Vandenbroucke J. P. (2007). The strengthening the reporting of observational studies in epidemiology (STROBE) statement: Guidelines for reporting observational studies. The Lancet.

[B43-ejihpe-16-00165] Wang T., Cheng H. (2025). Parenting stress and its influencing factors among chinese parents in parent–grandparent co-parenting families: A latent profile analysis. Behavioral Sciences.

[B44-ejihpe-16-00165] Wu P., Robinson C. C., Yang C., Hart C. H., Olsen S. F., Porter C. L., Jin S., Wo J., Wu X. (2002). Similarities and differences in mothers’ parenting of preschoolers in China and the United States. International Journal of Behavioral Development.

[B45-ejihpe-16-00165] Zhang C., Cubbin C., Ci Q. (2019). Parenting stress and mother–child playful interaction: The role of emotional support. Journal of Family Studies.

